# Drosophila Food-Associated Pheromones: Effect of Experience, Genotype and Antibiotics on Larval Behavior

**DOI:** 10.1371/journal.pone.0151451

**Published:** 2016-03-17

**Authors:** Julien Thibert, Jean-Pierre Farine, Jérôme Cortot, Jean-François Ferveur

**Affiliations:** Centre des Sciences du Goût et de l'Alimentation, UMR6265 CNRS, UMR1324 INRA, Université de Bourgogne Franche-Comté, 6, Bd Gabriel, 21000, Dijon, France; Alexander Fleming Biomedical Sciences Research Center, GREECE

## Abstract

Animals ubiquitously use chemical signals to communicate many aspects of their social life. These chemical signals often consist of environmental cues mixed with species-specific signals—pheromones—emitted by conspecifics. During their life, insects can use pheromones to aggregate, disperse, choose a mate, or find the most suitable food source on which to lay eggs. Before pupariation, larvae of several *Drosophila* species migrate to food sources depending on their composition and the presence of pheromones. Some pheromones derive from microbiota gut activity and these food-associated cues can enhance larval attraction or repulsion. To explore the mechanisms underlying the preference (attraction/repulsion) to these cues and clarify their effect, we manipulated factors potentially involved in larval response. In particular, we found that the (*i*) early exposure to conspecifics, (*ii*) genotype, and (*iii*) antibiotic treatment changed *D*. *melanogaster* larval behavior. Generally, larvae—tested either individually or in groups—strongly avoided food processed by other larvae. Compared to previous reports on larval attractive pheromones, our data suggest that such attractive effects are largely masked by food-associated compounds eliciting larval aversion. The antagonistic effect of attractive *vs*. aversive compounds could modulate larval choice of a pupariation site and impact the dispersion of individuals in nature.

## Introduction

To develop and reproduce, animals need to perceive multimodal sensory signals informing them on the quality of their environment and on the presence and reproductive status of conspecifics [1]. Both environmental and species-specific cues often combine their effect to elicit the behavioral response in the receiver animal [2, 3, 4].

Among the multiple sensory cues used to communicate, chemical signals, or pheromones, are ubiquitously used by animals [5, 6]. In insects, pheromones are involved in many social behaviors occurring from larval to adult life. In adults, they can modulate aggregation, mating and aggression [7, 8, 9]. Pheromones are also important during pre-adult development. For example, larval aggregation in gregarious species often relies on chemical cues mixed in feces [10, 11], whereas in non-social insects, behavior can be influenced by adult chemical cues [12, 13]. Reciprocally, larvae developing in the food can produce chemical cues affecting adult female attraction, oviposition and maternal care [14, 15].

In Drosophila, the effect of adult pheromones has been deeply investigated on adult behavior, especially in *D*. *melanogaster* that is amenable to genetic manipulation. In this species, adult pheromones can modulate adult aggregation [16], courtship, mating [17, 18] and aggression [19, 20, 21]. Drosophila pheromones can also affect larval behavior and pupariation. A field study revealed that some chemical cues produced by *D*. *simulans* and *D*. *buzzatii* species developing in the same fruit can change their pupariation site [22]. These cues, produced both by adults and larvae, induce a repulsive behavior in larvae, this potentially affecting their subsequent choice of a pupariation site [13]. Early developmental exposure to these cues also altered larval and pupal behaviors in the two species [22]. Among the varied compounds inducing larval aggregation on food sources already processed by other larvae, two pheromones were identified together with the neurons involved in their detection [23, 24, 25].

The yeast present on the food can enhance Drosophila response [14] whereas the microbiota hosted in their digestive system [26] can dramatically modulate the quantity and quality of the chemicals produced by both larvae and adults and therefore strongly impact their behavior. This is probably the reason why antibiotic-treated Drosophila flies showed a substantially altered mating behavior [27].

**Fig 1:**
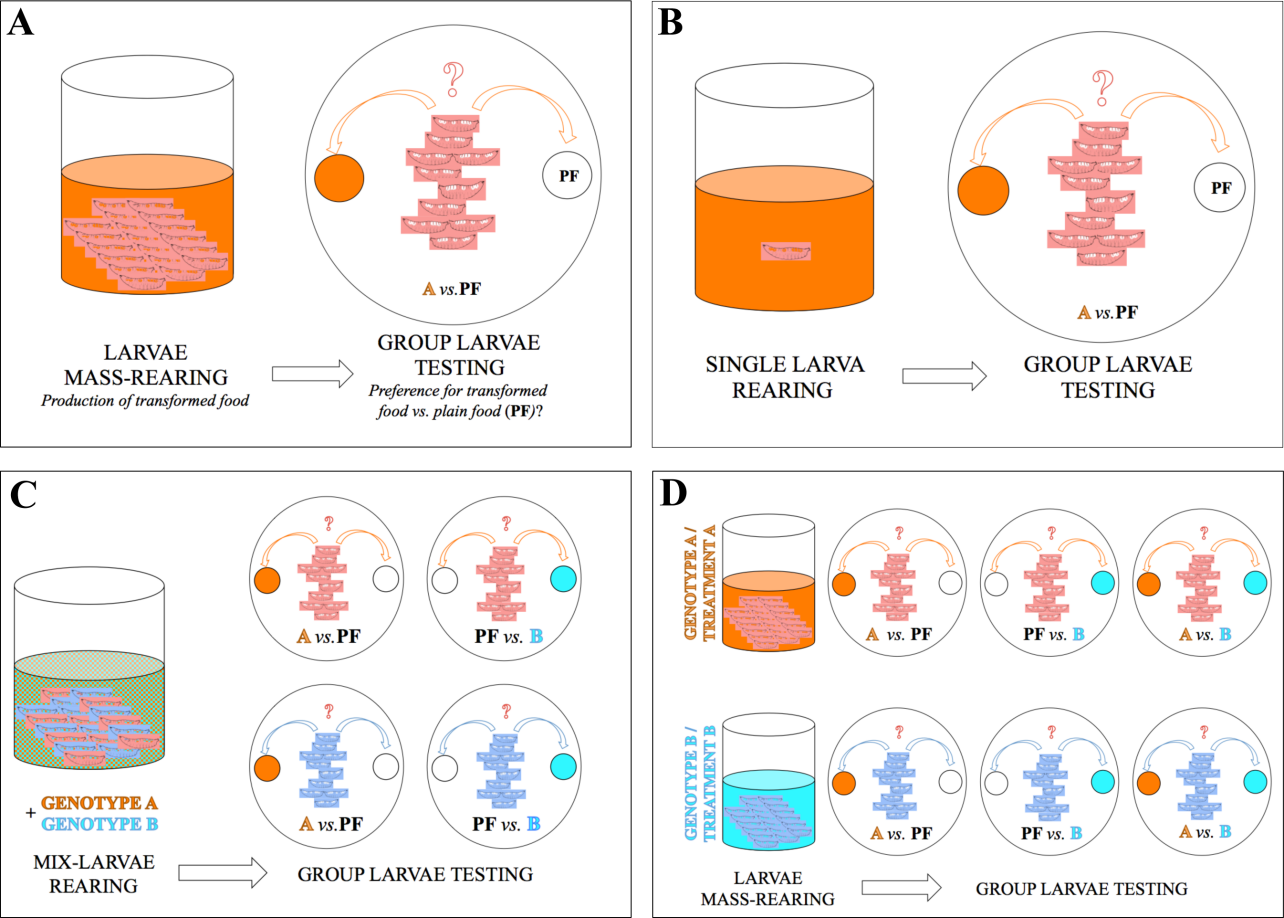
Schematic representation of the experimental procedures used to condition and test larvae. (**A**) In some experiments (shown on Figs 2, 3A and 4A), larvae were mass-raised and then tested by groups of 10 to a dual-choice involving plain food (PF; empty circle) *vs*. the food processed by these larvae (full colored circle). Note that the experiment involving mass-raised larvae tested individually is not shown (Fig 3B). (**B**) In one experiment (Fig 4B), larvae were individually raised and tested by groups of 10 to the same dual-choice test as in A. (**C**) In one experiment (Fig 7), larvae of two genotypes raised together (mix-larvae rearing) were sorted and tested by genotype to a choice associating PF with each processed food (food processed by genotype A or by genotype B). (**D**) In several experiments (Figs 5, 8, 9 and 10), larvae were assayed to food processed by their own genotype (or resulting of the same antibiotic treatment) or to food processed by other genotypes (or resulting of other treatments). In one experiment (Fig 6), larvae were tested to a choice of two larval-processed foods (A *vs*. B).

Here, we explored some of the mechanisms underlying the behavioral effect induced by larval pheromones in order to determine whether these cues have an attractive or a repulsive effect. We manipulated several biological factors potentially involved in this response, such as group-effect and the influence of early developmental exposure to larval chemicals (Fig 1). To generalize our findings, and assess the accuracy of larval response, we compared the response of two species and of several wild-type and transgenic strains, raised either separately or mixed together and we also measured the effect of antibiotics on larval and pupariation behaviors.

## Results

### A- Response in *D*. *melanogaster* Wild-Type Strains

#### 1- Canton-S strain: group tests

**Fig 2:**
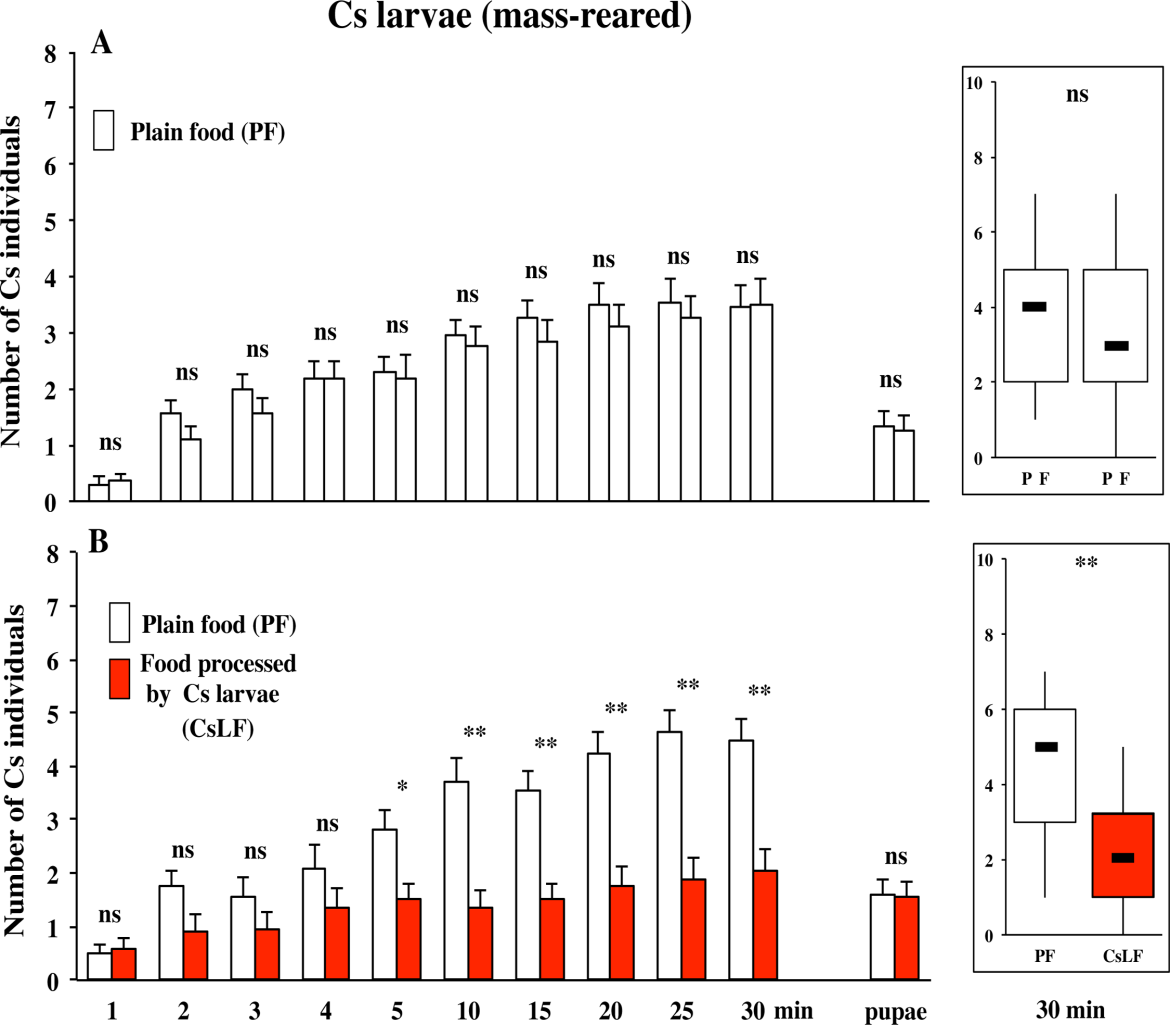
Food preference of Canton-S larvae in a dual choice test. Mass-reared early 3^rd^ instar Canton-S larvae (Cs) were tested by groups of 10 in a dual-choice test consisting of a petri dish containing two patches either impregnated with plain food (PF; empty bars) on both sides (**A**) or of a PF patch paired with a patch impregnated with food processed by Cs larvae (CsLF; filled bars; **B**). Their distribution on the two food patches during the observation period, was noted every minute between the start of the experiment and 5 min, and every 5 min until 30 min. The distribution of pupae on the two patches was also noted few days later (see right bars). Histograms show the mean (± sem) distribution of larvae and pupae on two food patches. On the right panel a simpler “box and whisker plots” representation is shown for the 30 min time point. The statistical difference for distribution between the two food patches was tested at each time point using a Wilcoxon test (at all indicated time points): **: p<0.01; *: p<0.05; ns: p>0.05. N = 20 groups/condition.

We first tested the response in groups of 10 larvae in the Canton-S (Cs) wild-type strain. Cs larvae, produced by a mass-rearing procedure, were given a two-food patches choice and their preference to either patch was measured during 30 min (Fig 1A). Their pupariation site was also determined, few days later. In the control test involving two plain food (PF) patches, a total of 70% Cs larvae—and later 20% pupae—aggregated on the patches without showing any preference (Fig 2A).

The experimental test consisted to pair one PF patch with a second patch impregnated with the food processed by Cs larvae (Cs larval food: CsLF). After 5 min of test, Cs larvae showed a preference for PF (and avoided CsLF), and this marked difference increased with time (Fig 2B). At 30 min, 67% larvae aggregated on both food patches with a clear preference to PF compared to CsLF (45.7 and 21.3%, respectively), whereas pupae were equally distributed on the two food patches. This indicates that Cs larvae, but not pupae, avoid the food labeled with their own food-derived compounds.

#### 2- Dijon2000 strain: group and individual tests

**Fig 3:**
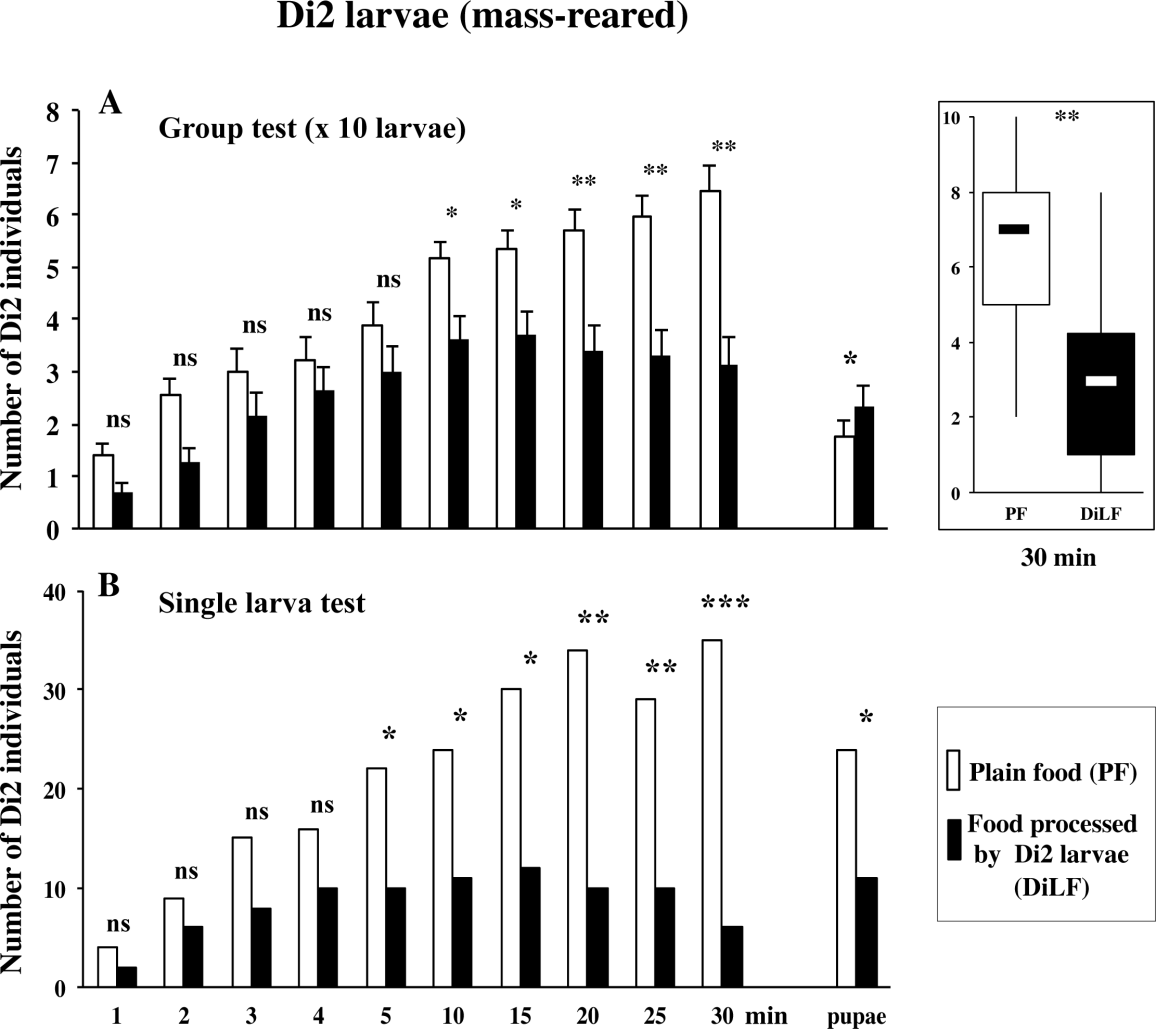
Food preference of Dijon2000 larvae tested either in groups or individually. Mass-reared early 3^rd^ instar Dijon2000 larvae (Di2) were tested either by groups of 10 (**A**) or individually (**B**) in a dual-choice test consisting of a petri dish containing two patches: one impregnated with plain food (PF; empty bars), and the other with food processed by Di2 larvae (DiLF; filled bars). The measurements were carried as in Fig 2. Histograms show the mean (± sem) distribution or the box and whisker plots representation at 30 min (A), and the number of larvae and pupae (B) on each patch. These distributions were tested at each time point using a Wilcoxon test (A) or a binomial Poisson distribution (B). The statistical significance is indicated as follows: ***: p<0.001; **: p<0.01; *: p<0.05; ns: p>0.05. N = 20 groups (A), or 100 larvae (B).

To generalize this finding, we assayed larvae of a second wild-type strain, Dijon2000 (Di2). Mass-reared Di2 larvae tested in groups (of 10) showed no preference in the PF/PF control assay (data not shown) whereas in the experimental dual choice-test associating PF with DiLF (LF processed by Di2 larvae), they clearly preferred PF over DiLF (at 30 min: 64 and 24%, respectively; p<0.01; Fig 3A).

To detect for a potential group effect, we performed tests with individual Di2 larvae (also produced by a mass-rearing procedure). In the “PF/PF” control test, 84% larvae migrated on either PF patch without showing any preference (data not shown) whereas in the experimental “PF/DiLF” test, individual Di2 larvae preferred PF (p<0.001; Fig 3B). Larvae first oriented more frequently to the PF patch (N = 68) than the DiLF patch (N = 12; Two-tailed Poisson binomial test: p = 0.0107) but they took the same time to reach either patch (378±49 and 266±43 sec, respectively; Mann-Whitney test: Z = 1.1209; p = 0.263). Also, individual larvae spent more time on the PF patch than on the DiLF patch (1133±91 and 721±108 sec, respectively; Mann-Whitney test: Z = 2.737; p = 0.0061). Also, more individuals pupariated on the PF (47%) than on the DiLF (22%) patches, differently to Di2 tested in groups (Fig 3A). These data show that Di2 larvae, similarly to Cs larvae, are strongly repulsed by their own food-derived compounds. For the next tests, we preferentially used the Di2 strain whose larval global response was higher than in Cs strain. For the sake of clarity, we only show the larval distribution at 30 min represented as box and whisker plots. The detailed data indicating the dynamic response together with the pupariation site choice can be found in the Supporting Information section.

### B- Effect of Early Experience

**Fig 4:**
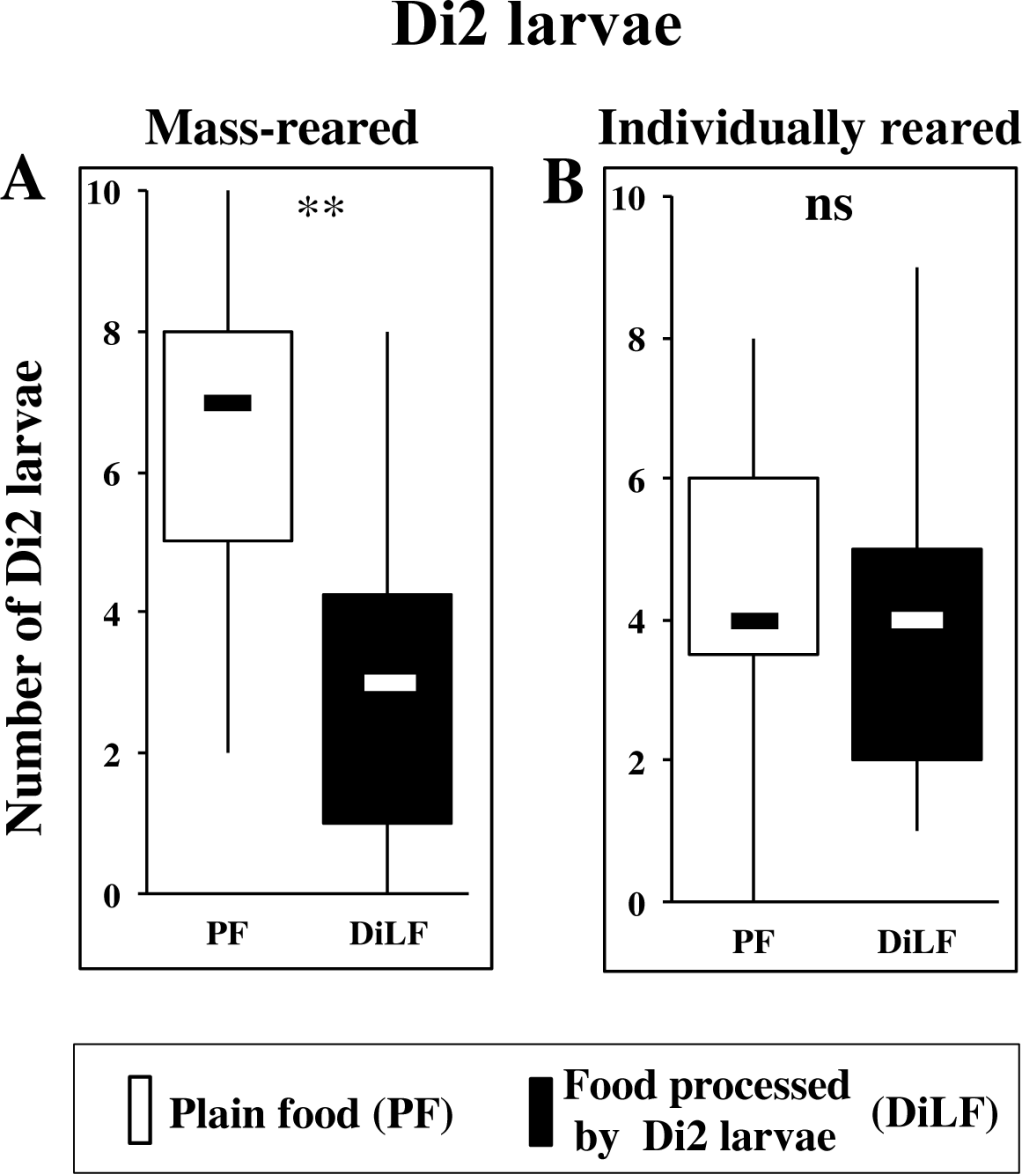
Food preference of Dijon2000 larvae raised either communally or individually. Third instar Di2 larvae either produced by mass rearing procedure (**A**) or individually raised (**B**) were tested by groups of 10, and their distribution (and that of pupae) were noted on the two types of food (PF = empty bars; Di2 processed food or DiLF = filled bars) as in Fig 3A. Box and whisker plots show the distribution of larvae at 30 min in each experiment. The statistical difference for distribution between the two food patches was tested using a Wilcoxon test: **: p<0.01; *: p<0.05; ns: p>0.05. N = 20 (A) and 35 (B) groups.

Based on earlier results obtained with other Drosophila species [22], we checked the effect of early developmental exposure to conspecifics. We raised Di2 larvae individually on fresh food (since their early embryonic development) and subsequently tested them by groups (of 10). The total number of larvae aggregating on food patches at 30 min was similar in the two raising conditions (87±5%; Fig 4). However, differently from mass-reared larvae (Fig 4A), larvae raised in isolation showed no food preference (Figs 1B and 4B; S1 Fig). In either case, no preference was detected in pupae. This experiment indicates a clear effect of the early exposure to larval-processed food.

### C- Intra- and Interspecific Variations

#### 1- Intra-specific variation

**Fig 5:**
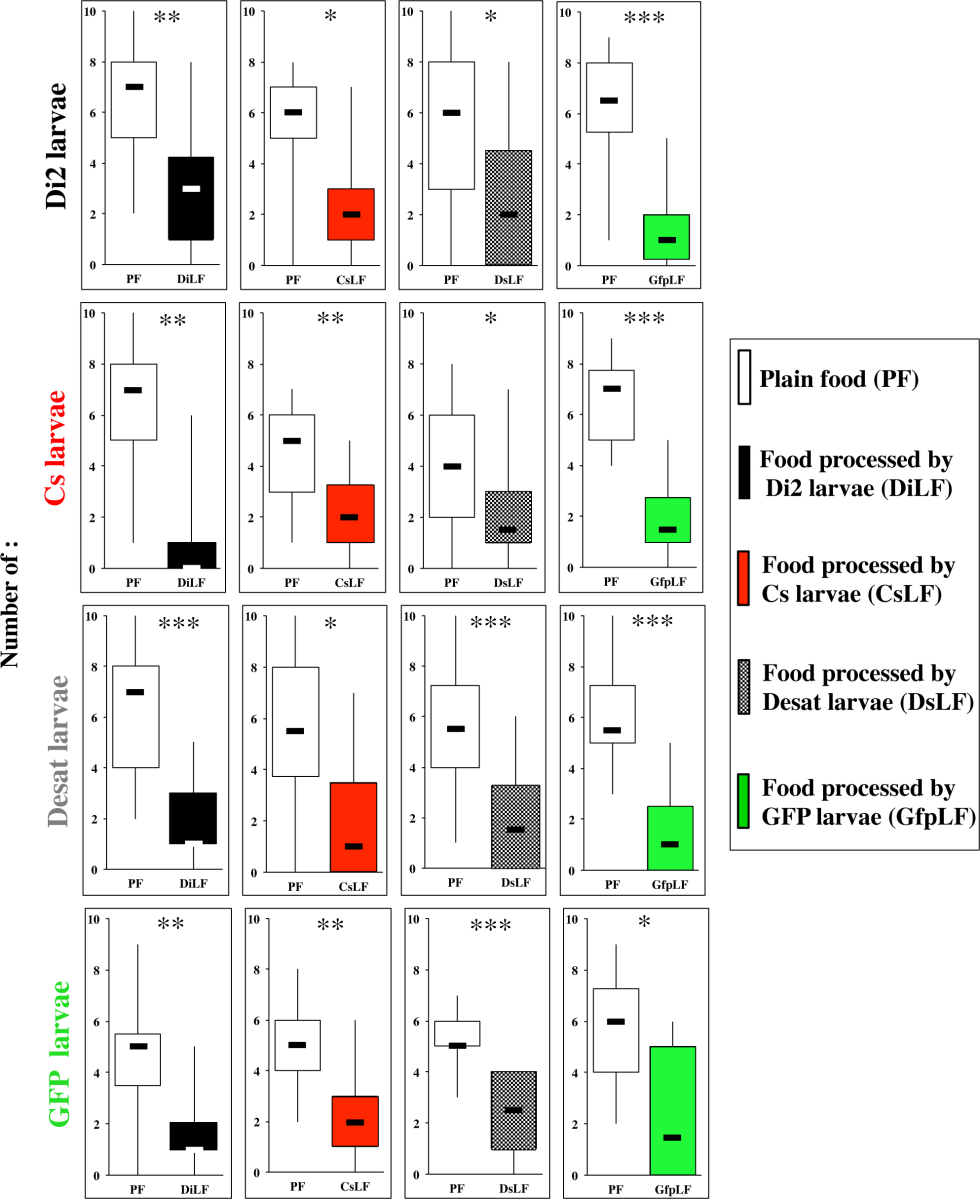
Food preference of *D*. *melanogaster* larvae of four strains to the food processed by these four strains. The food preference of third instar larvae of two wild type strains (Di2, Cs; two top rows) and of two *desat1* mutant lines (Desat, GFP; two bottom rows) was tested in dual-choice test associating PF (empty bars) with food processed by larvae of these four strains. The food types correspond to columns: from left to right: food processed by Di2 (DiLF), Cs (CsLF), Desat (DsLF) and GFP (GfpLF) larvae (The same color code was used to indicate the genotype of larvae and the food they had processed). Each experiment was carried as in Fig 2, and the statistics were also performed using a Wilcoxon test. The statistical significance is indicated as follows: ***: p<0.001; **: p<0.01; *: p<0.05; ns: p>0.05. N = 15–30 groups.

Our next aim consisted to search potential effects related to population-specific variation with regard to production of food-derived chemical cues. To test this, we compared the response of four strains: two wild-type strains (Cs and Di2), and two *desat1* mutant lines altered for fatty acid metabolism (Fig 5). The original *desat1-1573* strain is homozygous for an insertion in the *desat1* gene (Desat). This is also the case for the *desat1-GFP* mutant line which additionally contains the UAS-GFP reporter transgene (GFP; useful for larva sorting; see next section). We assayed all 16 possible larval x food combinations to evaluate the behavioral effects related to (*i*) each larval experience, and (*ii*) the behavioral effect induced by food-derived compounds. Therefore, the four larvae (Di2, Cs, Desat, GFP = four rows of Fig 5) were presented to a dual choice-test always combining PF with each of the four LFs respectively processed by these larvae (DiLF, CsLF, DsLF, GfpLF = four columns of Figs 1D and 5). All larvae showed a preference for PF with some slight dynamic variation (S2 Fig). This indicates the general repulsive effect of larval-processed food either tested to their own strain (diagonal items on Fig 5) or to other strains.

**Fig 6:**
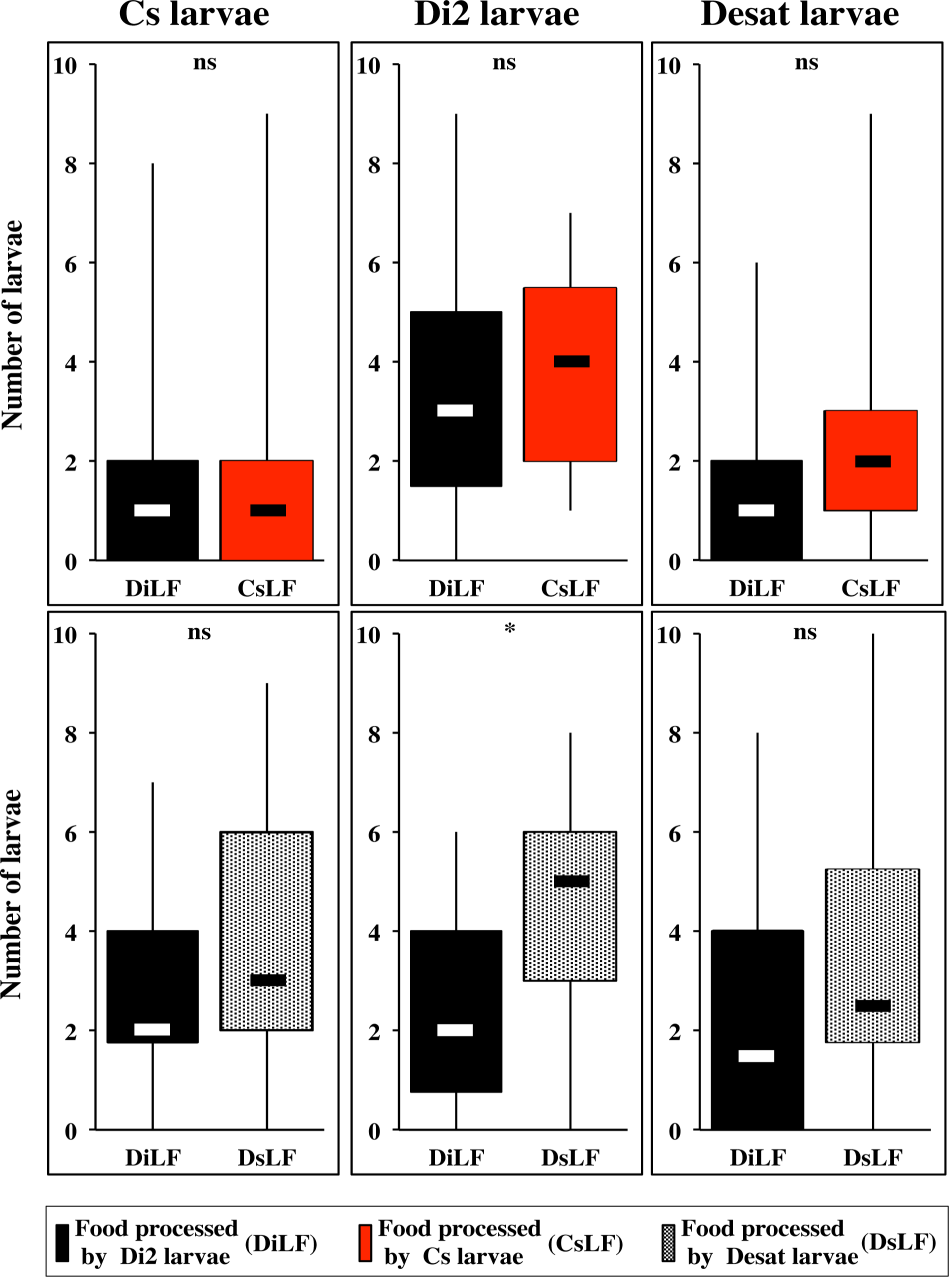
Food preference of *D*. *melanogaster* larvae of three strains in dual-choice tests combining two types of larval-processed food. The food preference of third instar larvae of two wild-type strains (Di2, Cs; two left columns) and the *desat1* mutant line (Desat; right column) was tested to a dual-food choice combining two types of larval processed foods (PF was not used here). The top series of box plots represent the dual-food choice combining the food processed by Di2 (DiLF) with the food processed by Cs larvae (CsLF); the bottom series combines DiLF with the food processed by Desat larvae (DsLF). For parameters and statistics, please refer to Fig 2. The statistical difference for distribution between the two food patches was only significant for Di2 larvae tested in the “DiLF/DsLF” choice: (*: p<0.05). All other tests showed no difference (ns: p>0.05). N = 18–20 groups.

To detect more subtle larval discrimination ability between compounds processed by different strains, Cs, Di2 and Desat larvae were given a simultaneous choice of two LFs (Figs 1D and 6). No preference was observed, except for Di2 larvae which slightly preferred DsLF over DiLF, at 30 min (p<0.05). Note the overall low number (<35%) of Cs and Desat larvae on CsLF/DiLF patches after 30 min (S3 Fig). This indicates that Di2 larvae show an acute discrimination of LFs and that when two LFs are simultaneously used, they induced an increased repulsive effect on larvae.

#### 2- Mixed population cultures

**Fig 7:**
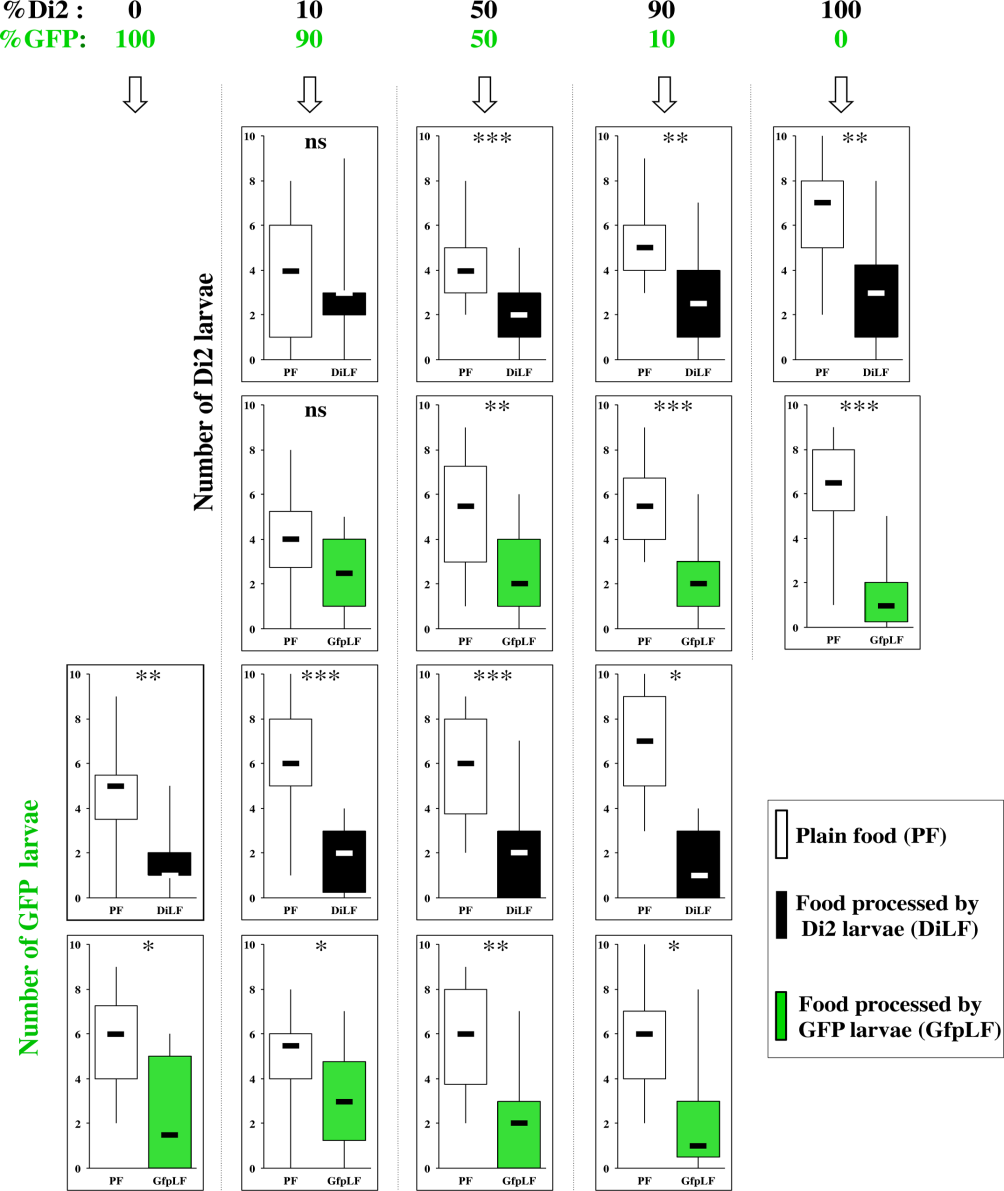
Food preference in *D*. *melanogaster* larvae of two strains cultured together. Individuals of the Di2 and GFP strains were cultured together in different ratio (see at the top of each column of data: from left to right: 0/100, 10/90, 50/50, 90/10 and 100/0) starting from the egg development (100 eggs/culture). The distribution of third instar larvae of the two genotypes (raised in these conditions) was measured in two dual-choice experiments: (*i*) PF associated with Di2-processed food (DiLF) and (*ii*) PF associated with GFP-processed food (GfpLF). Only one genotype could be tested in each of the two “pure” culture conditions (100% GFP on the left; 100% Di2 on the right). For parameters and statistics, please refer to Fig 5. N = 10–20 groups.

Based on the ability of Di2 larvae to discriminate between DsLF and DiLF (Fig 7), we further explored the mechanistic origin of this difference. Based on the clear early food-exposure effect (Fig 4) [22], we attempted to quantitatively change the amount of population-specific compounds. Practically, we raised Di2 and GFP larvae together in different proportions. Then, we measured the preference of each co-cultured larvae in a dual choice associating PF with either DiLF or GfpLF (the GFP fluorescent larvae were separated from the Di2 larvae under UV light; Fig 1C). The two « pure » populations were used as controls (for GFP larvae, see left row data below 0% Di2 / 100% GFP; for Di2 larvae, see right row data below 100% Di2 / 0% GFP; Fig 7; S4 Fig). Most Di2 and GFP larvae raised in mixed cultures (with the 10/90, 50/50 and 90/10 ratios) showed a clear PF preference after 30 min. Only Di2 larvae raised in the 10/90 ratio (10% Di2 / 90% 1573-GFP) showed no PF preference in both tests. Therefore, when larvae of two genotypes were raised together, their subsequent food preference could be altered depending upon their respective frequency.

#### 3- Intra- and interspecific responses

**Fig 8:**
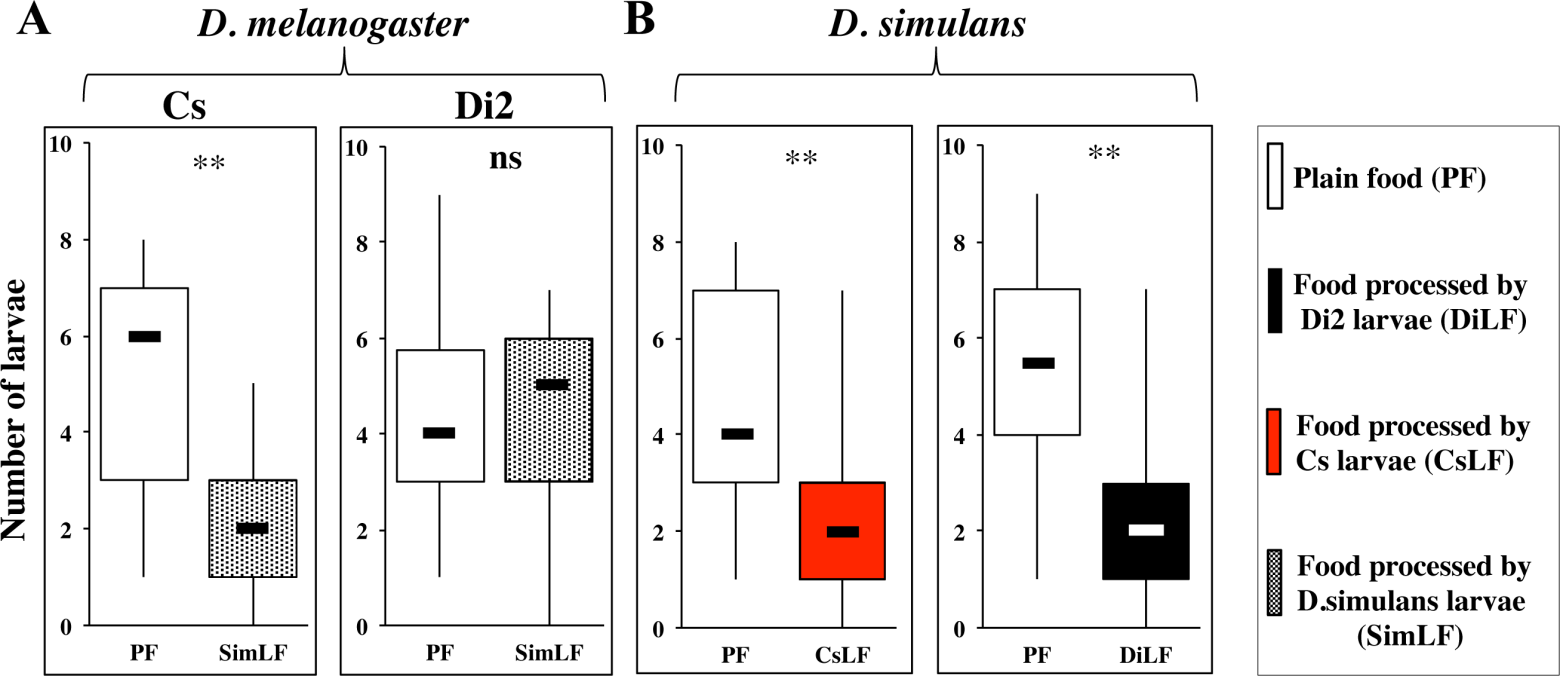
Food preference in *D*. *melanogaster* and *D*. *simulans* larvae in intra- and interspecific assays. The food preference of third instar larvae of two *D*. *melanogaster* wild-type strains (Di2, Cs) and of a *D*. *simulans* wild type strain were reciprocally assayed. The box and whisker plots show the response (**A**) of each *D*. *melanogaster* strain to a choice between PF and *D*. *simulans*-processed food (SimLF), and (**B**) of *D*. *simulans* to PF associated with food processed by each *D*. *melanogaster* strain (for Cs: CsLF; for Di2: DiLF). For parameters and statistics, please refer to Fig 5. N = 20 groups.

Based both on the Cs *vs*. Di2 intraspecific variation (Fig 6; S3 Fig) and on the relative conservation of larval pheromones between *D*. *melanogaster* and *D*. *simulans* [25], we performed cross-experiments between the two *D*. *melanogaster* strains and one *D*. *simulans* strain. More precisely, we measured the responses (*i*) of Cs and Di2 larvae to *D*. *simulans* LF (SimLF) and reciprocally (*ii*) of *D*. *simulans* larvae to CsLF or to DiLF (Fig 8; S5 Fig). Interestingly, Cs larvae, but not Di2 larvae, preferred PF over SimLF (Fig 8A) whereas, in the reciprocal experiment, *D*. *simulans* larvae preferred PF over both CsLF and DiLF (Fig 8B). This behavioral difference indicates that the production and/or perception of food-derived compounds vary between both *D*. *melanogaster* strains.

### D- Effect of Antibiotics

**Fig 9:**
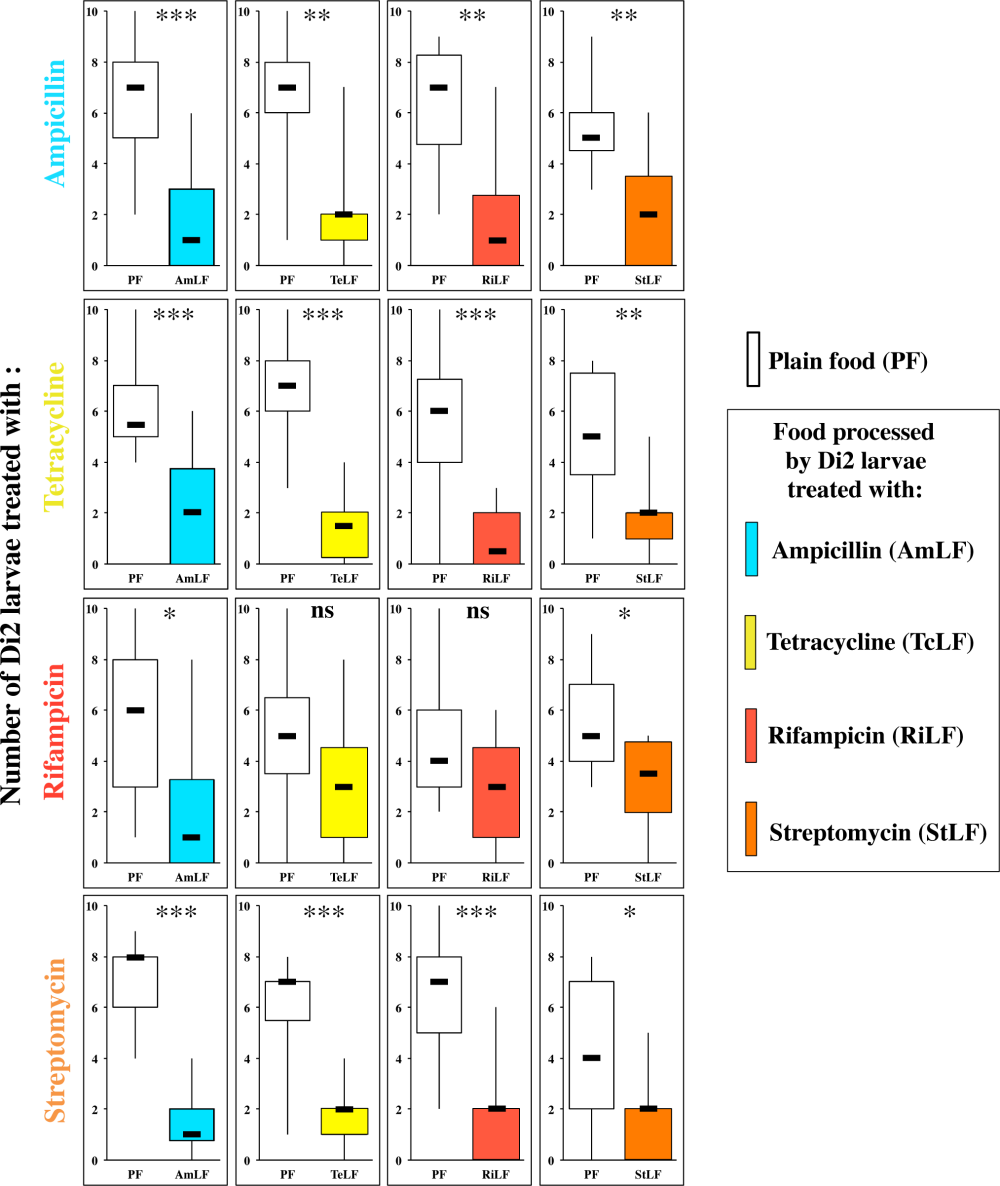
Food preference in Dijon2000 larvae treated with four antibiotics to food produced by these four treated larvae. The food preference of third instar Di2 larvae raised in PF added with the four antibiotics (Ampicillin, Tetracycline, Rifampicin, Streptomycin) was assayed. Each group of treated Di2 larvae (respectively shown as the four rows from top to bottom) was assayed in a dual-choice test associating each type of food processed by these treated larvae (from left to right; AmLF, TeLF, RiLF, StLF; see color code) with PF (empty bars). For parameters and statistics, please refer to Fig 5. N = 15–25 groups.

**Fig 10:**
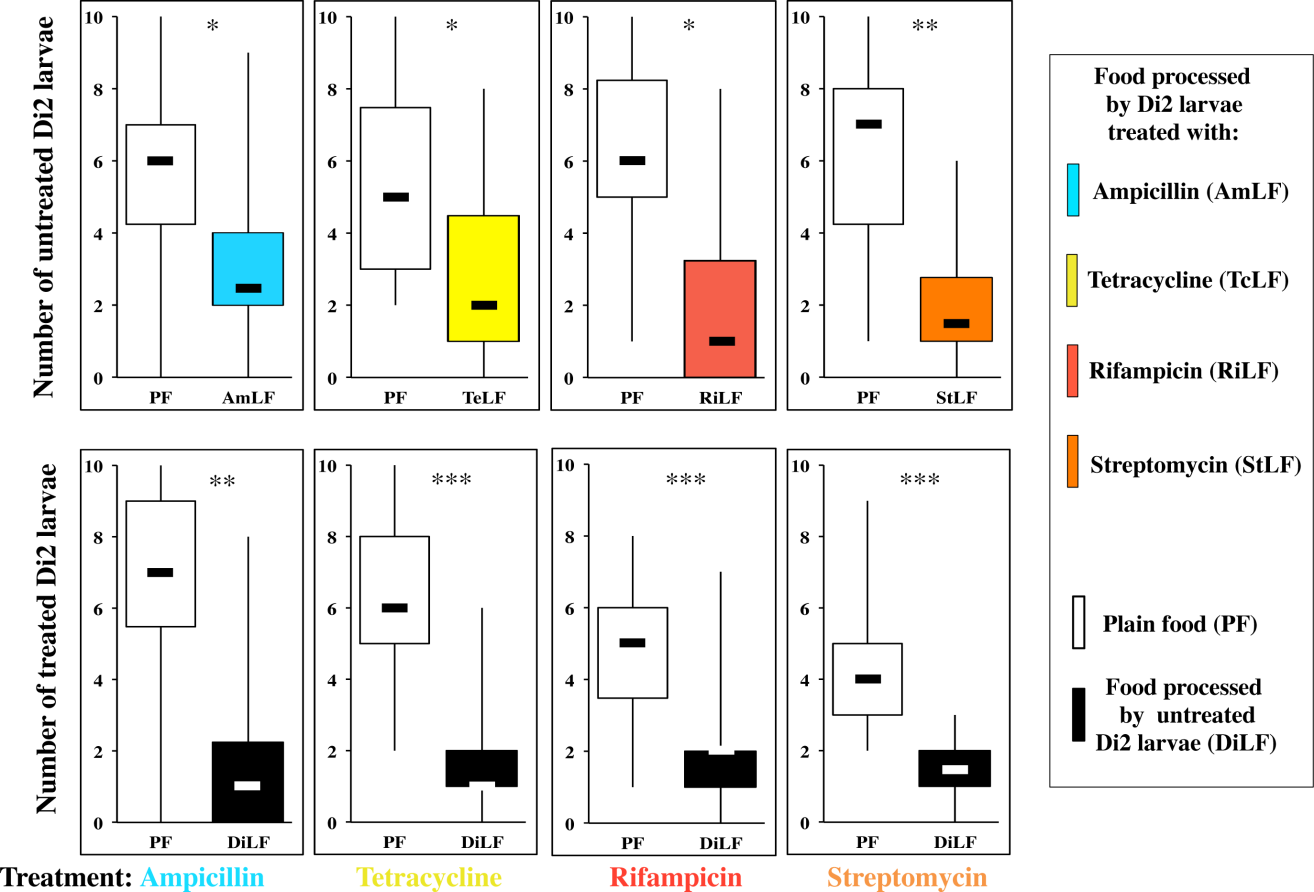
Control experiments of food preference in Dijon2000 larvae treated with four antibiotics to food produced by these four treated larvae. To control the effect of the antibiotic treatment (*i*) on the food quality and (*ii*) on the response of Di2 treated larvae, we measured (*i*) the larval response of untreated Di2 larvae to the four types of food processed by Di2 larvae treated with four antibiotics (see top row of box plots; from left to right: Ampicillin = AmLF, Tetracycline = TeLF, Rifampicin = RiLF, Streptomycin = StLF; the color code is similar as in Fig 9 associated with PF (empty bars), and (*ii*) the response of Di2 larvae respectively treated with the same four antibiotics (bottom row of box plots; see tretment below bars, from left to right) in dual-choice tests always associating PF with Di2 larvae-processed food (DiLF; filled bars). For parameters and statistics, please refer to Fig 5. N = 10–20 groups.

Given the effect induced by antibiotic treatment on adult behavior [27], we investigated the behavioral effect of Ampicillin, Tetracycline, Rifampicin and Streptomycin during pre-adult development. These substances are know to affect different types of bacteria (Gram-positive and -negative bacteria; www.sigmaaldrich.com). We separately measured whether each of these four antibiotics added in the food could affect the behavior of such treated Di2 larvae (four rows in Fig 9; S6 Fig) in response to the four LFs respectively processed by these treated larvae (four columns in Fig 9; S6 Fig: AmLF, TeLF, RiLF and StLF). In most experimental tests, larvae clearly preferred PF over LFs. However, in Rifampicin-treated larvae, this preference decreased (in tests involving PF against AmLF or StLF) or disappeared (PF *vs*. TeLF or RiLF). Differently, larval PF preference was not altered in all control tests either involving (*i*) Di2 larvae presented to the four treated LFs, or (*ii*) the four antibiotic-treated larvae presented to DiLF (Fig 10; S7 Fig; see also Fig 1D). Therefore, antibiotics can induce very different effects, and more specially Rifampicin, with regard to the production and/or perception of larval food-derived compounds.

## Discussion

### General Effect of Food-Derived Pheromones on Larval Behavior

Our principal goal consisted to measure larval food preference to food-derived molecules, some of which acting as pheromones [13], and to determine whether larvae were attracted or repulsed by such compounds. Moreover, we explored some of the mechanisms underlying both the production and behavioral response to larval compounds. Overall, we found that most larvae preferred plain food (PF) whereas they avoided larval processed food (LF). The fact that the total number of larvae present on the two food patches was similar in the control (PF/PF) and in the experimental tests (PF/LF) indicates that a similar number of larvae responded to either food patch whereas the difference of distribution between the two patches was largely due to the compounds present in most LFs inducing a repulsive effect. The total number of larvae dramatically decreased when two LFs were combined indicating a enhanced aversive effect induced by two patches (Fig 6). Larval aversion against LF started after 5 to 10 min in wild-type larvae tested to wild-type processed LFs, or several minutes later with some manipulated larvae and/or LFs. The number of larvae on LF patches was generally stable after 5 min whereas the number on PF patches increased until 15 to 30 min (S1–S7 Figs). The repulsive effect of LF was not due to a potential group-effect bias, at least in the Di2 strain (Fig 3) [28].

### Divergence with Previous Reports

Strikingly, the repulsive effect on larvae of LF-associated compounds found here diverges from the attractive response described in previous reports [22, 24, 25]. Such discrepancy can be partly explained by the divergence of methods and parameters. It appears that *D*. *simulans* and *D*. *buzzatii* larvae individually tested [22]) differently responded than when tested in groups [13] (this report; Fig 8). Although no group-effect affected the response of the *D*. *melanogaster* Di2 strain (Fig 3), we cannot exclude the possibility that a similar effect exists in other Drosophila species or populations. A second study measuring the initial attraction in single larva and the food consumed by groups during several hours reported a higher performance on LF [24]). Differently, “our” larvae which showed more initial attraction on PF than to LF, did not show faster migration to either patch. This seems to support the olfactory effect of LF-associated cues [29] revealing, in our case, that these cues did not induce an attractive effect but rather a repulsive one. A third study performed with groups of larvae reported an attractive effect for two larval pheromones [(*Z*)-5- and (*Z*)-7-tetradecenoic acids: 5TD and 7TD, respectively] when separately tested on agarose [25]. However, given that when the two compounds were combined, they induced no attraction, it is thus possible that their interaction (eventually in addition to other food-derived compounds not present in agarose but in plain food) induced a different effect. Indeed, the complex food-derived mixture induced a clear aversive effect ([13]; this report). This indicates that in our experiments, the larval attractive effect of 5TD or of 7TD (separately tested) is partially masked by the global repulsive effect induced by other food-derived compounds additionally the PF context.

### Effect of Experience

Similarly to *D*. *simulans* and *D*. *buzzatii* species [22], the preference of *D*. *melanogaster* larvae was altered depending on their early exposure to their own transformed food. Another report showed a “learning” effect enhancing larval attraction to their own labeled food [24]. Differently, in our hands, early exposure did not induce attraction, but rather suppressed repulsion and/or induced indifference ([30]; Fig 4). Given that the relative behavioral valence between the two experiments varied in the same direction (increased attractiveness = decreased aversiveness), the absolute difference between both experiments may be only due to the food context (see above). Moreover, some of our effects could be also caused by social interaction. Since we did not dissociate the « chemical » from the « social » informations, we cannot exclude this possibility. However, the fact that Di2 larvae (raised in the 10/90 ratio with GFP larvae; Fig 7; S4 Fig) were not repulsed by LFs suggests that larval aversive behavior normally relies more on chemical than on social information. Based on this, we believe that the difference observed between mass- and individually-reared larvae was simply due to the quantitative variation of food-derived compounds released in the food by larvae. This means that exposure to a low amount of chemicals (produced by a single larvae) was not sufficient to induce an effect differently to that induced by mass-reared larvae which produced—and were exposed to—higher amounts of these compounds. Whatever factor(s) is (are) involved, the clear avoidance of mass-reared larvae to their own compounds demonstrates the strong effect of prior exposure to these compounds. Together, our results indicate that larval migration depends on a mixture of compounds that can either induce attractive or repulsive effects, the combination of which induces food preference. Early developmental exposure to the repulsive cues may be more potent than exposure to the attractive cues, this explaining why the early exposure of Di2 larvae to some treatments (such as Rifampicin; Fig 9; see below) induced no subsequent, or less, larval repulsion to LFs.

### Effect of Genotype and Microbiota

Beside this clear “early-exposure effect”, our other experiments show that the use of different populations or antibiotic treatments can differently alter larval behaviors. This may result of a variable developmental impregnation induced by different food-derived compounds.

We also report intraspecific difference between wild-type strains (Cs and Di2; Figs 6 and 8) or wild-type and transgenic strains (Fig 7). In particular, the asymetric “loss” of discrimination of 10% Di2 larvae—but not of GFP larvae—raised with 90% larvae of the other genotype indicates a variation for food-derived compounds between the two strains.

Populations (or genotypes) of a same species can also vary for their microbiota profile [26], this largely impacting the quality and quantity of food-derived compounds. The selective elimination of some bacteria clearly affects larval attraction [31], and this may explain the contrasted effects induced by our different antibiotic treatments. In particular, the altered response of Rifampicin-treated larvae and pupae may result of an abnormal production of aversive compounds that are normally perceived and learned by larvae during their early development. This may explain why larvae and pupae, not imprinted by these compounds, have lost their preference to PF (or their aversion against LF). Given that larvae treated with the three other antibiotics, as well as control larvae, showed a normal response to RiLF, this suggests that these compounds should be associated with other(s) chemical(s) to allow a correct associative learning process [29].

### Ecological Relevance

We do not know yet whether the neurological mechanisms underlying this effect fit with those described for other types of larval learning [32]. Such learning process may involve the association between food and food-derived compounds, or pheromones, as in mammals [33]. In Drosophila, the food-derived pheromones produced by developing larvae could increase the salience of some food components subsequently associated with these pheromones. Early developmental chemosensory exposure could also change the adult preference to similar food compounds. This was observed with pure fatty acids which, after early larval exposure, changed female food preference for egg-laying behavior [34]. This is relevant to our study since larvae produce several fatty acids in LF [13]. Moreover, the larva-to-adult persistence of the « food-pheromone » preference could be transmitted to the next generation if females resulting of exposed larvae continuously lay eggs on the same preferred substrate. This effect, iteratively repeated generation after generation, could promote the selection of some of the genes underlying « food-pheromone » chemosensory preference, this explaining some tight plant-insect relationships.

## Material and Methods

### Strains

Drosophila strains were raised in 150 ml glass vials containing 50ml of yeast/cornmeal/agar medium and kept in a breeding room at 24.5±0.5°C with 65±5% humidity on a 12:12h light/dark cycle (subjective day from 8:00am to 8:00pm). Flies were transferred every two/three days to avoid larval competition and to regularly provide abundant progeny for testing. All behavioral experiments were performed under similar conditions.

We used two *D*. *melanogaster* wild-type strains: Canton-S (Cs), a old-established strain widely used in fly laboratories, and Dijon 2000 (Di2), a strain maintained in our lab for 15 years which showed very stable behavioral performances.

We also used two transgenic strains: *desat1-1573-Gal4* (abbreviated as Desat) and *desat1-1573-Gal4; UAS-CD8-GFP* (abbreviated “1573-GFP” or “GFP” in the text). The two strains are homozygous for a P-Gal4 element inserted in the *desat1* gene regulatory region [35] potentially affecting larval metabolism [36] with potential consequence on the production of food-derived compounds. Additionally to the P-Gal4 elements, the 1573-GFP strain also contains two copies of the *UAS-CD8-GFP* reporter transgene which shows fluorescent expression in the tissues where *desat1-1573*-Gal4 is expressed [37].

The *D*. *simulans* strain (gift of Prof. Raùl Godoy-Herrera, Santiago, University of Chile) was derived from multi-female lines originated from fruits collected in Chile [22] and maintained in our laboratory for more than four years before testing.

### Behavior

#### Preparation of experiments

Two hours before the experiment, the food patches to be tested (Whatman paper grade 42, 1.5 cm diameter, pre-washed in distilled water, in ethanol, and dried overnight at 70°C) were impregnated either with plain food (PF), or with food processed by larvae of mixed ages (1^st^ instar (L1) to 3^rd^ instar (L3)) resulting of mass mating (larval food = LF). Adults were rapidly removed after egg-laying. Therefore, apart larval cues, LFs could contain chemical traces left by adults [13]. However, we can exclude the effect of adult cues on larval repulsion since the mixed-culture tests involving the transfer of eggs to fresh PF showed the same global aversive effect (Fig 7).

#### Behavioral parameters

Tests were always performed with early L3 individuals, between 12:30 and 17:00, under white light at 24.5±0.5°C. Just before the test, L3 were separated from the food and maintained in distilled water. Tests were carried out in freshly prepared Petri dishes (9.5 cm diameter, 1 cm high) containing a 2% water agar layer (thickness 5 mm). Each Petri dish contained two food patches (separated by approximately 40 mm and placed in diametrically opposed zones) impregnated with various food types (see above). Food-patches—from which excess food was removed with a spatula—were separately pinned down on the agar dish with a thin needle. Depending on the test, a group of ten larvae, or a solitary larva, was transferred, using a fine brush, at a mid-distance between the patches. Then, the dish was covered with a lid to reduce evaporation of the tested media and the time of observation started. For the “individual larva” experiment, the time to reach the first chosen patch and the duration spent on the patch was noted. For group experiments, the number of larvae present on each patch was noted each min for the first 5 min of the experiment, and then every 5 min until 30 min. The 30 min duration was determined based on a previous report [13]. Petri dishes containing assayed larvae were further kept at 24.5±0.5°C for the next two days and the number of pupae on each patch was noted.

In control experiments, we tested the response to a pair of patches impregnated with P-food. In other experiments, we either paired one P-food patch with one “impregnated-food” patch, or two impregnated-food patches. Control and “impregnated-food” experiments were simultaneously performed.

#### Statistics

For each group experiment, we assessed the statistical difference for larval (and pupal) distribution between both food patches using a Wilcoxon test (XLStats) with the R® sofware. For individual tests, we used the binomial Poisson distribution test. In the principal section, and for the sake of clarity, we only show the larval distribution on the two food patches at 30 min, as box and wisker plots. In the Supporting Information section, we show the distribution at all time-points (+ pupae) to better visualize the dynamic response of larvae, but for the experiments involving many conditions, we only show four time-points (3, 5, 15 and 30 min) as representative data points. These histograms show the mean (± sem) of 10–31 experiments except for individual larva tests (N = 100) and always correspond to pooled experiments obtained on several days.

### Manipulation of Biological Factors

#### Social deprivation

In most experiments, we tested individuals issued from mass-reared culture except in one experiment (Fig 4B) where we tested the response of larva raised in isolation. In this case, freshly laid eggs (4±1 hours) were individually deposited in a fresh food vial and kept until early L3 stage for behavioral testing.

#### Mixed cultures

For mixed cultures associating Di2 and *desat1-1573*-GFP larvae, a controlled number of eggs of each genotype was deposited in a fresh food vials following the 10/90, 50/50 and 90/10 ratio to obtain a overall number of 100 egg/vial. Experiments involving “pure” Di2 and *desat1-1573*-GFP strains were also performed following our standard procedure (Fig 1C). Few hours before the test, larvae were sorted by genotypes according to the presence/absence of fluorescence in their body. This separation was carried out (without anaesthesia) with a fine brush under a Leica stereomicroscope MZ12 equipped with a fluorescent light emitter.

#### Antibiotic treatments

Antibiotic food was prepared with 100 ml PF initially put to a boil and then cooled down before addition of each antibiotic (present as a powder). For this amount of plain food, we used either 50mg Ampicillin, 20mg Rifampicin, 10mg Streptomycin ou 5mg Tetracycline (Sigma). Di2 females were allowed to lay eggs on each antibiotic-rich food and two days later, they were removed out of the vials. These four antibiotics were chosen based on (*i*) their effect on Drosophila adult behavior [27] and (*ii*) their complementary spectrum of bactericide effect (in particular for their antimicrobial activity against gram-positive and -negative bacteria; www.sigmaaldrich.com).

## Supporting Information

S1 Fig Food preference of Dijon2000 larvae (Di2) raised either communally or individually. Third instar Di2 larvae either produced by mass-rearing procedure (A) or individually raised (B) were tested by groups of 10, and their distribution (and that of pupae) were noted on the two types of food (PF = empty bars; DiLF = filled bars) as in Fig 3A. Histograms show the mean (± sem) distribution of larvae and pupae in each experiment. The statistical difference for distribution between the two food patches was tested at each time point using a Wilcoxon test (at all indicated time points): **: p<0.01; *: p<0.05; ns: p>0.05. N = 20 (A) and 35 (B) groups. The data shown here correspond to Fig 4.
(PDF)

S2 Fig Food preference of *D*. *melanogaster* larvae of four strains to the food processed by these four strains. The food preference of third instar larvae of two wild type strains (Di2, Cs; two top rows) and of two *desat1* mutant lines (Desat, GFP; two bottom rows) was tested in dual food-choice test associating PF (empty bars) with food processed by larvae of these four strains. The food types correspond to columns: from left to right: food processed by Di2 (DiLF), Cs (CsLF), Desat (DsLF) and GFP (GfpLF) larvae (The same color code was used to indicate the genotype of larvae and the food they processed). Each experiment was carried as in Fig 2, and the statistics were also performed using a Wilcoxon test. The statistical significance is indicated as follows: ***: p<0.001; **: p<0.01; *: p<0.05; ns: p>0.05. N = 15–30 groups. The data shown here correspond to Fig 5.
(PDF)

S3 Fig Food preference of *D*. *melanogaster* larvae of three strains in dual-choice tests combining two types of larval-processed food. The data shown here correspond to Fig 6.
(PDF)

S4 Fig Food preference in *D*. *melanogaster* larvae of two strains cultured together. Individuals of the Di2 and GFP strains were cultured together in different ratio (see at the top of the histograms. The distribution of third instar larvae (and pupae) of the two genotypes (raised in these conditions) was measured in two food-choice experiments: PF associated with Di2-processed food (DiLF) or PF associated with GFP-processed food (GfpLF). The data shown here correspond to Fig 7.
(PDF)

S5 Fig Food preference in *D*. *melanogaster* and *D*. *simulans* larvae in intra- and interspecific assays. The food preference of third instar larvae of two *D*. *melanogaster* wild-type strains (Di2, Cs) and of a *D*. *simulans* wild type strain were reciprocally assayed. The data shown here correspond to Fig 8.
(PDF)

S6 Fig Food preference in Dijon2000 larvae treated with four antibiotics to food produced by these four treated larvae. The food preference of third instar larvae of Di2 larvae raised in PF added with the four antibiotics (Ampicillin, Tetracycline, Rifampicin, Streptomycin) was assayed. Each group of treated Di2 larvae (respectively shown as the four rows from top to bottom) was assayed in a dual-choice test associating each type of food processed by these treated larvae (from left to right; AmLF, TeLF, RiLF, StLF; see color code) with PF (empty bars). For parameters and statistics, please refer to Fig 5. N = 15–25 groups. The data shown here correspond to Fig 9.
(PDF)

S7 Fig Control effect of antibiotic treatments in the food preference of Di2 larvae. To control the effect of the antibiotic treatment on the food quality, and on the response of Di2 treated larvae, we measured (*i*) the larval (and pupal) response on untreated Di2 larvae to the four types of food processed by Di2 larvae treated with four antibiotics (see top row of histograms, from left to right) Ampicillin (AmLF), Tetracycline (TeLF), Rifampicin (RiLF), Streptomycin (StLF; the color code is similar as on Fig 7) associated with PF (empty bars), and (*ii*) the response of Di2 larvae respectively treated with the same four antibiotics (bottom row of histograms; from left to right) in dual-choice tests always associating PF with Di2 larvae-processed food (DiLF; filled bars). For parameters and statistics, please refer to Fig 5. N = 10–20 groups. The data shown here correspond to Fig 10.
(PDF)
