# Supplementary figures and images for: Drosophila Food-Associated Pheromones: Effect of Experience, Genotype and Antibiotics on Larval Behavior
Source: PLoS One. 2016 Mar 17;11(3):e0151451. doi: 10.1371/journal.pone.0151451 (PMC4795598; doi:10.1371/journal.pone.0151451)

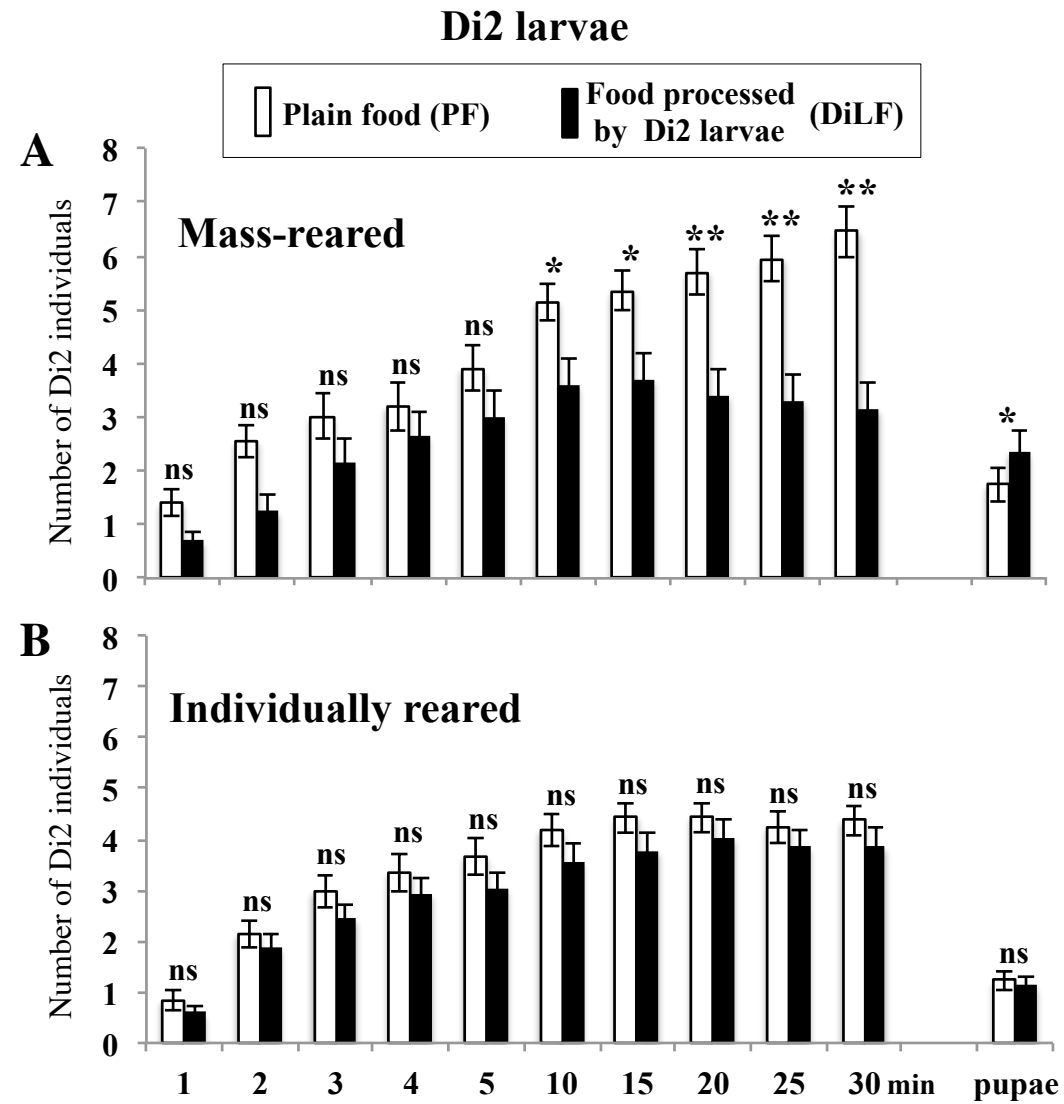

S1 Figure

Supplement: S1 Fig — Third instar Di2 larvae either produced by mass-rearing procedure (A) or individually raised (B) were tested by groups of 10, and their distribution (and that of pupae) were noted on the two types of food (PF = empty bars; DiLF = filled bars) as in Fig 3A. Histograms show the mean (± sem) distribution of larvae and pupae in each experiment. The statistical difference for distribution between the two food patches was tested at each time point using a Wilcoxon test (at all indicated time points): **: p<0.01; *: p<0.05; ns: p>0.05. N = 20 (A) and 35 (B) groups. The data shown here correspond to Fig 4. (PDF) [file pone.0151451.s001.pdf]

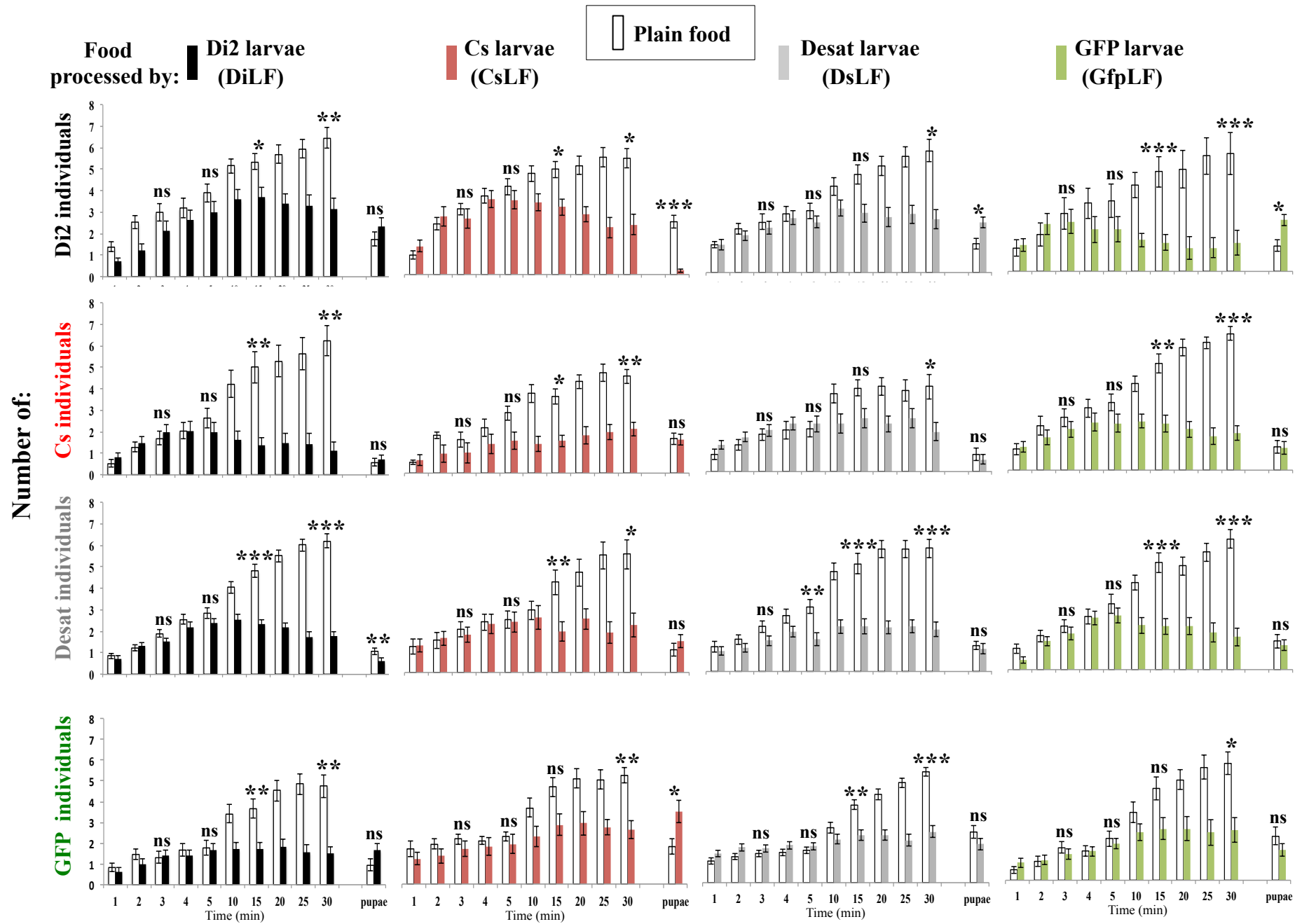

S2 Figure

Supplement: S2 Fig — The food preference of third instar larvae of two wild type strains (Di2, Cs; two top rows) and of two desat1 mutant lines (Desat, GFP; two bottom rows) was tested in dual food-choice test associating PF (empty bars) with food processed by larvae of these four strains. The food types correspond to columns: from left to right: food processed by Di2 (DiLF), Cs (CsLF), Desat (DsLF) and GFP (GfpLF) larvae (The same color code was used to indicate the genotype of larvae and the food they processed). Each experiment was carried as in Fig 2, and the statistics were also performed using a Wilcoxon test. The statistical significance is indicated as follows: ***: p<0.001; **: p<0.01; *: p<0.05; ns: p>0.05. N = 15–30 groups. The data shown here correspond to Fig 5. (PDF) [file pone.0151451.s002.pdf]

Food processed by:  Di2 larvae (DiLF)  Cs larvae (CsLF)  Desat larvae (DsLF)

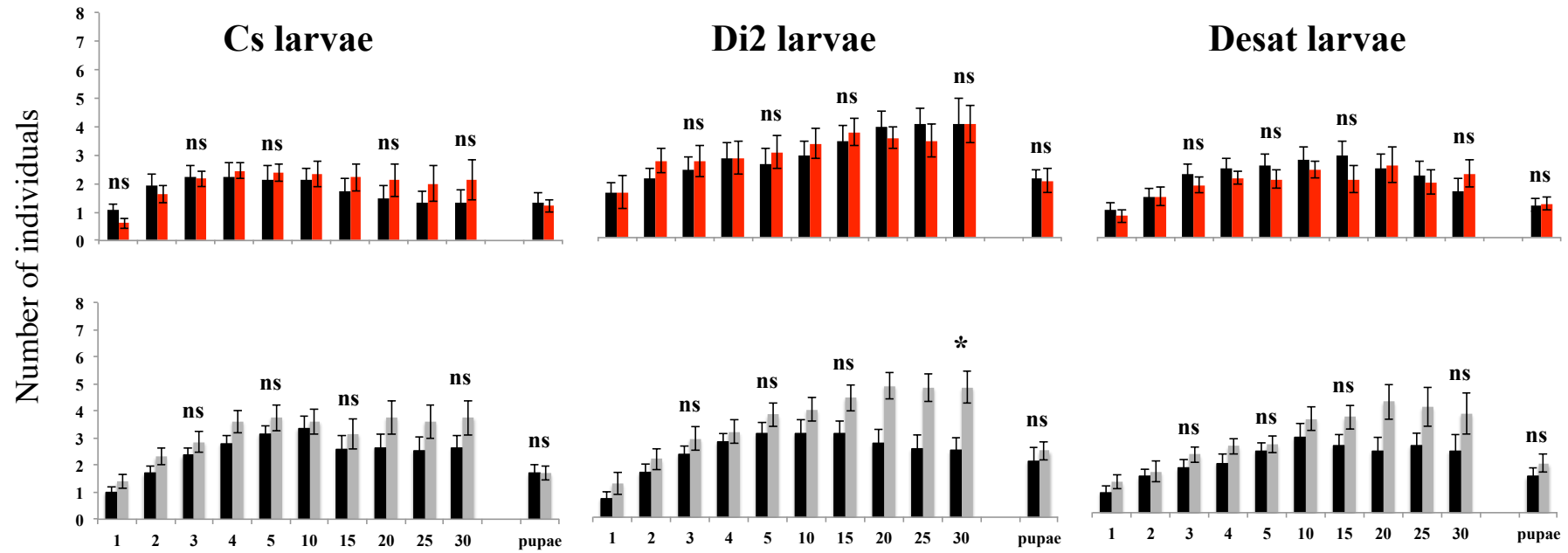

S3 Figure

Supplement: S3 Fig — The data shown here correspond to Fig 6. (PDF) [file pone.0151451.s003.pdf]

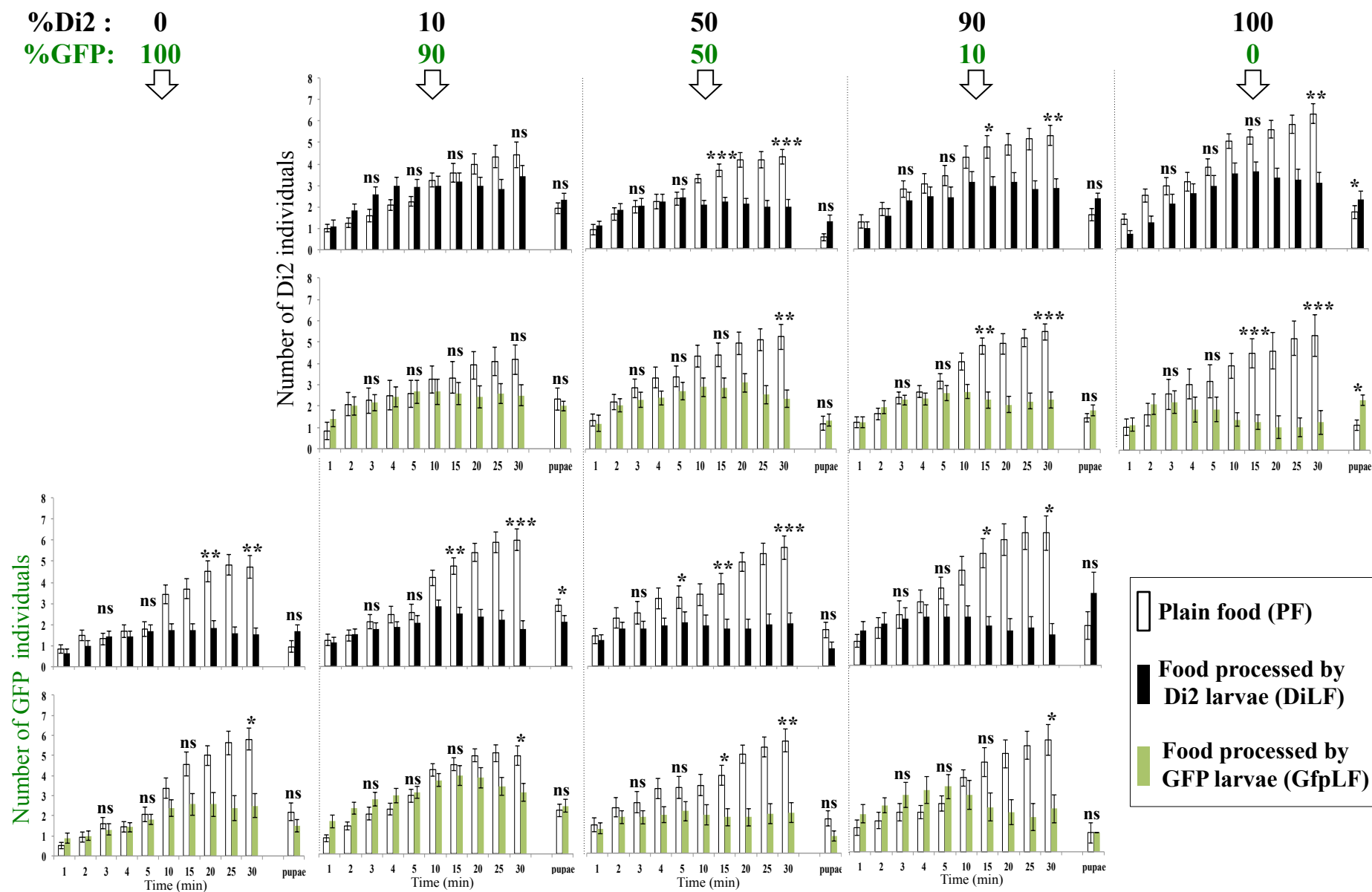

S4 Figure

Supplement: S4 Fig — Individuals of the Di2 and GFP strains were cultured together in different ratio (see at the top of the histograms. The distribution of third instar larvae (and pupae) of the two genotypes (raised in these conditions) was measured in two food-choice experiments: PF associated with Di2-processed food (DiLF) or PF associated with GFP-processed food (GfpLF). The data shown here correspond to Fig 7. (PDF) [file pone.0151451.s004.pdf]

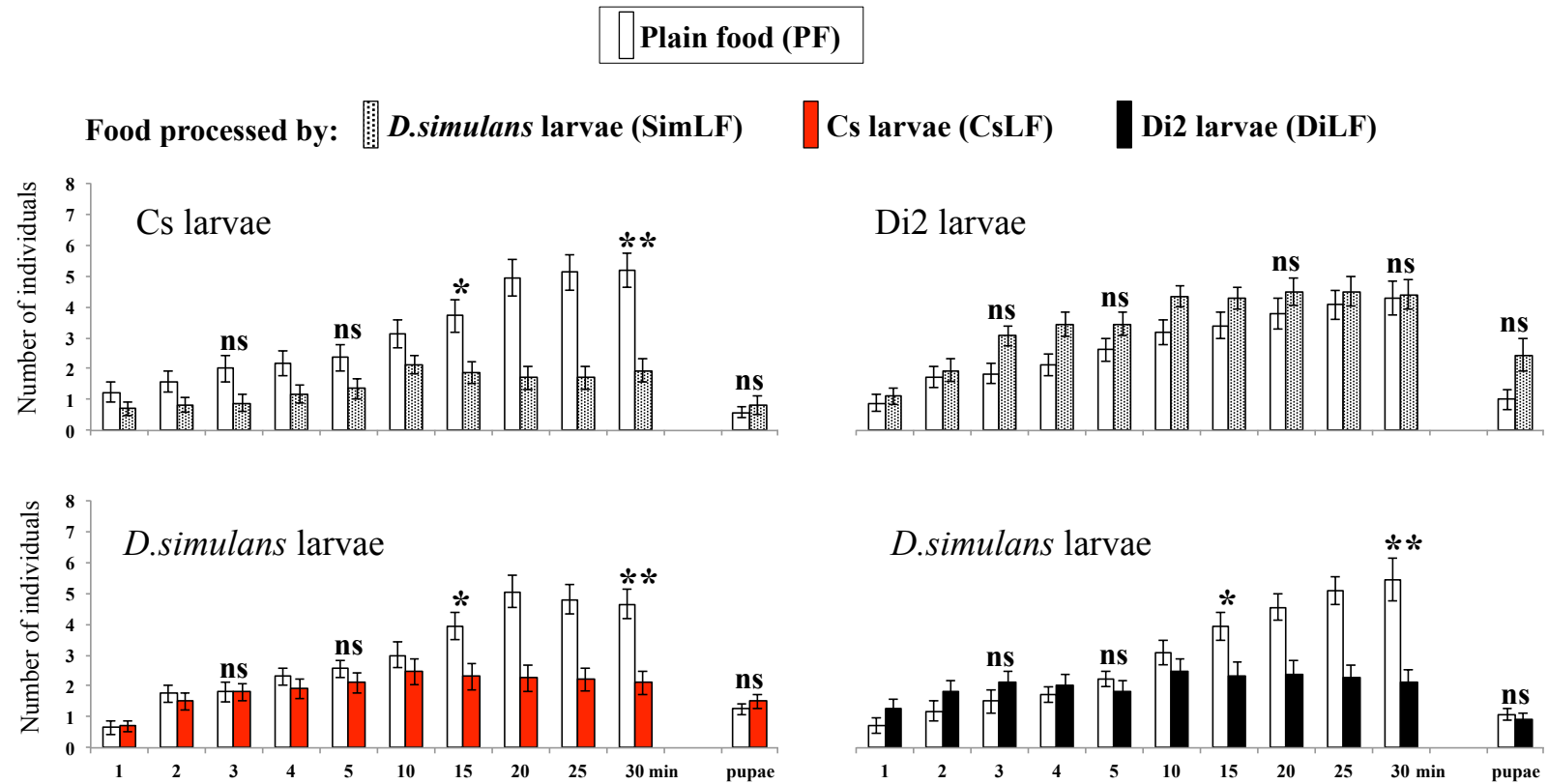

S5 Figure

Supplement: S5 Fig — The food preference of third instar larvae of two D. melanogaster wild-type strains (Di2, Cs) and of a D. simulans wild type strain were reciprocally assayed. The data shown here correspond to Fig 8. (PDF) [file pone.0151451.s005.pdf]

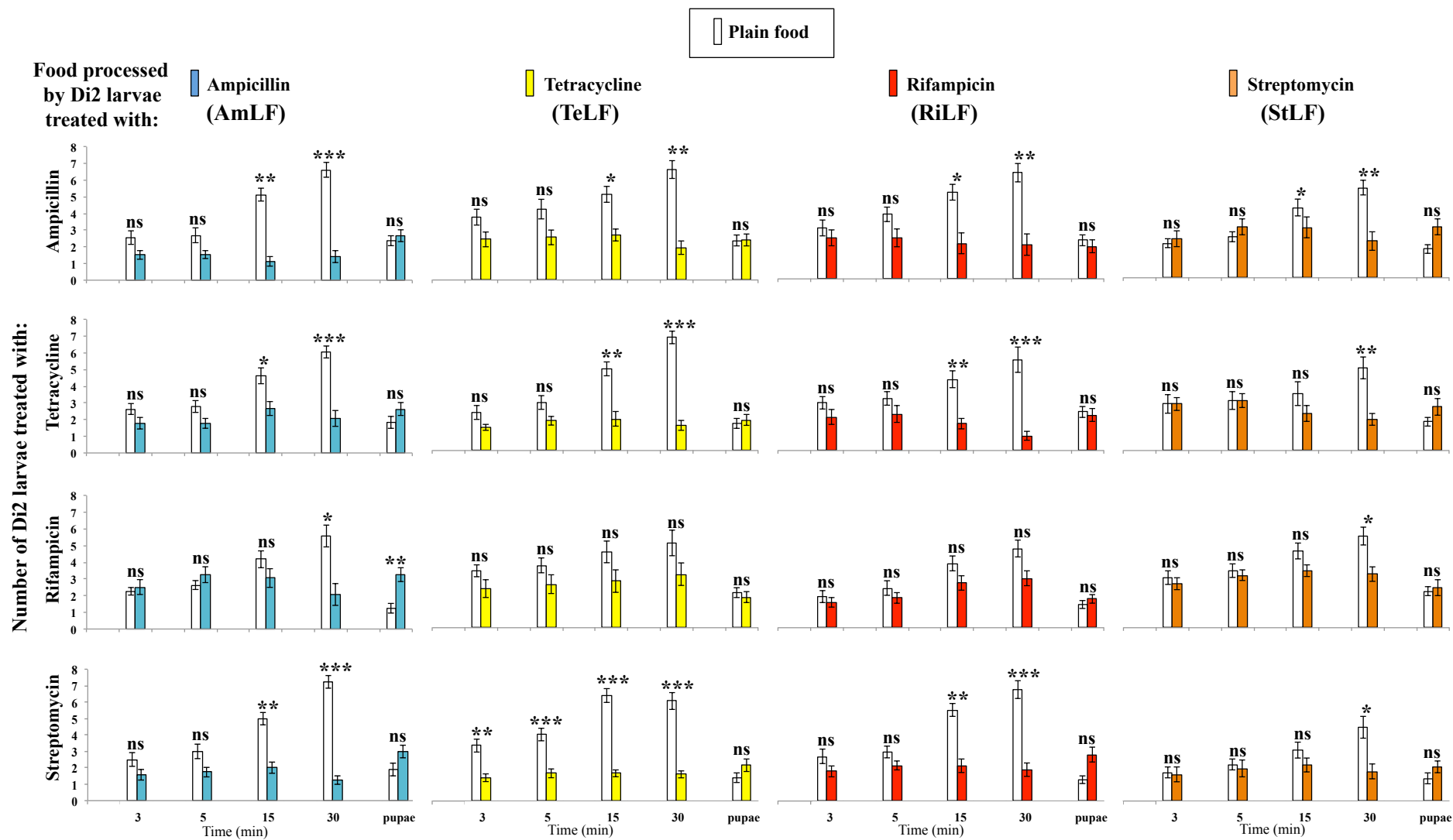

S6 Figure

Supplement: S6 Fig — The food preference of third instar larvae of Di2 larvae raised in PF added with the four antibiotics (Ampicillin, Tetracycline, Rifampicin, Streptomycin) was assayed. Each group of treated Di2 larvae (respectively shown as the four rows from top to bottom) was assayed in a dual-choice test associating each type of food processed by these treated larvae (from left to right; AmLF, TeLF, RiLF, StLF; see color code) with PF (empty bars). For parameters and statistics, please refer to Fig 5. N = 15–25 groups. The data shown here correspond to Fig 9. (PDF) [file pone.0151451.s006.pdf]

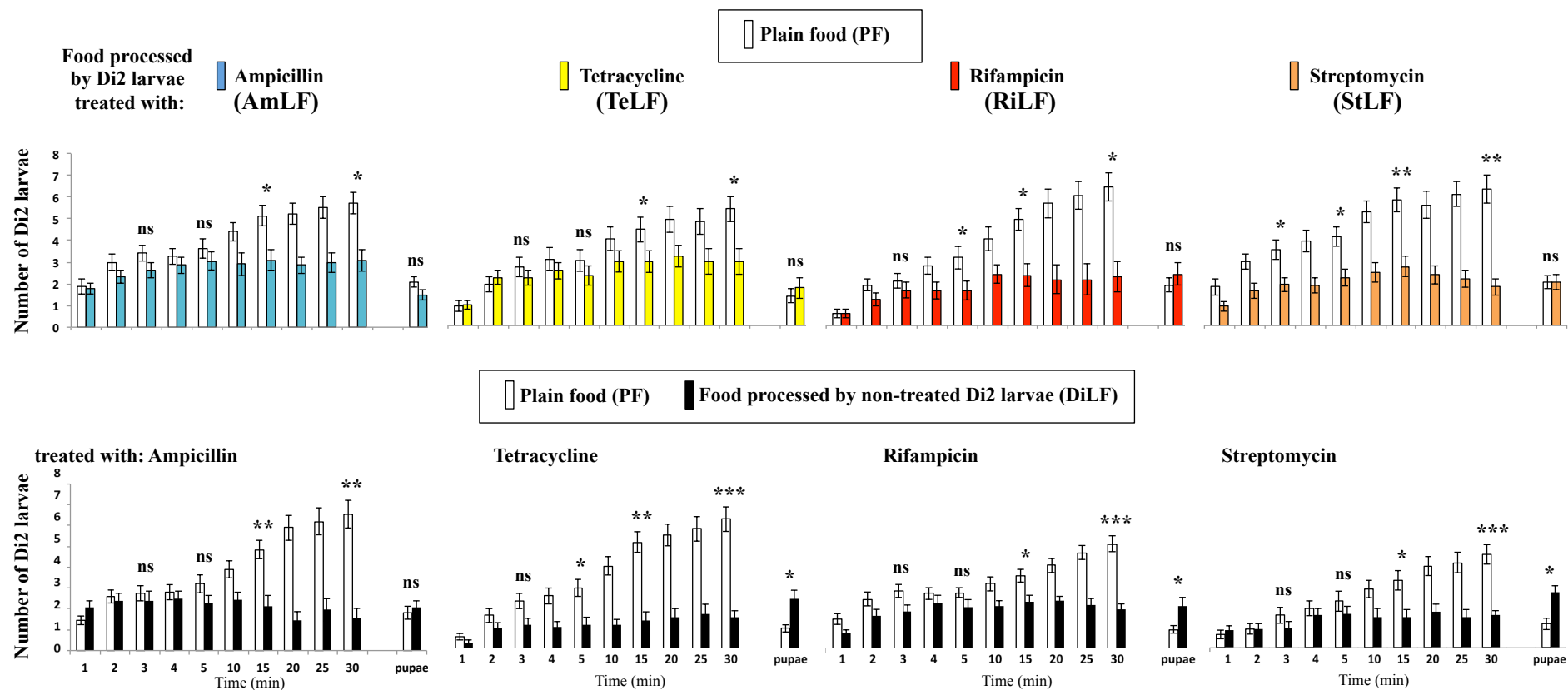

S7 Figure

Supplement: S7 Fig — To control the effect of the antibiotic treatment on the food quality, and on the response of Di2 treated larvae, we measured (i) the larval (and pupal) response on untreated Di2 larvae to the four types of food processed by Di2 larvae treated with four antibiotics (see top row of histograms, from left to right) Ampicillin (AmLF), Tetracycline (TeLF), Rifampicin (RiLF), Streptomycin (StLF; the color code is similar as on Fig 7) associated with PF (empty bars), and (ii) the response of Di2 larvae respectively treated with the same four antibiotics (bottom row of histograms; from left to right) in dual-choice tests always associating PF with Di2 larvae-processed food (DiLF; filled bars). For parameters and statistics, please refer to Fig 5. N = 10–20 groups. The data shown here correspond to Fig 10. (PDF) [file pone.0151451.s007.pdf]
